# Simple action for depression detection: using kinect-recorded human kinematic skeletal data

**DOI:** 10.1186/s12888-021-03184-4

**Published:** 2021-04-22

**Authors:** Wentao Li, Qingxiang Wang, Xin Liu, Yanhong Yu

**Affiliations:** 1 School of Computer Science and Technology, Qilu University of Technology (Shandong Academy of Sciences), Jinan, China; 2 College of Traditional Chinese Medicine, Shandong University of Traditional Chinese Medicine, Jinan, China

**Keywords:** Depression detection, Machine learning, Kinect sensor, Human skeleton joints

## Abstract

**Background:**

Depression, a common worldwide mental disorder, which brings huge challenges to family and social burden around the world is different from fluctuant emotion and psychological pressure in their daily life. Although body signs have been shown to present manifestations of depression in general, few researches focus on whole body kinematic cues with the help of machine learning methods to aid depression recognition. Using the Kinect V2 device to record participants’ simple kinematic skeleton data of the participant’s body joints, the presented spatial features and low-level features is directly extracted from the record original Kinect-3D coordinates. This research aimed to constructed machine learning model with the preprocessed data importing, which could be used for depression automatic classification.

**Methods:**

Considering some patients’ conditions and current status and refer to psychiatrists’ advices, simple and significant designed stimulus task will lead human skeleton data collection job. With original Kinect skeleton data extracting and preprocessing, the proposed experiment demonstrated four strong machine learning tools: Support Vector Machine, Logistic Regression, Random Forest and Gradient Boosting. Using the precision, recall, sensitivity, specificity, roc-curve, confusion matrix et.al, indicators were calculated as the measurement of methods, which were commonly used to evaluate classification methodologies.

**Results:**

Across screened 64 pairs with age and gender totally matching in depression and control group, and Gradient Boosting achieved the best performance with the prediction accuracy of 76.92%. Sorted by female (54.69%) and male for the gender-based depression recognition, we applied best performance classifier Gradient Boosting got prediction accuracy of 66.67% in the male group, and 71.73% in the female group. Utilizing the best model Gradient Boosting for age-based classification, prediction accuracy got 76.92% in the older group (age >40, 50% of total) and 53.85% accuracy in the younger group (age <= 40).

**Conclusion:**

The depression and non-depression individuals can be well classified by computational models using Kinect captured skeletal data. The Gradient Boosting, an excellent machine learning tool, get the performance in the four methods we demonstrated. Meanwhile, in the gender-based depression classification also gets reasonable accuracy. In particular, the recognition results of the old group are significantly better than that of the young group. All these findings suggest that kinematic skeletal data based depression recognition can be applied as an effective tool for assisting in depression analysis.

## Background

Depression is a common worldwide mental disorder, which is different from fluctuant emotion and psychological pressure in their daily life, and brings huge challenges to family and social burden around the world [1, 2]. Depression has become a serious health condition, especially patient symptoms long-lasting and with moderate or severe intensity, because it may cause the affected human to suffer strongly and foundation function poorly at work, at school and in family. [3, 4] Major Depressive Disorder (MDD) represents a leading cause of disability worldwide and a significant cost to health care systems. However, depressive symptoms are difficult to measure, especially cognitive decline, which may lead to suicide without timely diagnosis and treatment in the worst. [5–7]

Questionnaires capturing depressive symptoms had been the most commonly used and showed great success in psychiatric practice [8]. Clinical depression diagnosis [9, 10] will measure the presence of markedly diminished interest or pleasure, combined with at least four of the following symptoms for a period exceeding two weeks, i.e. fatigue or loss of energy almost, sleeping disturbances, diminished ability to concentrate or indecisiveness in their daily life [11]. By providing an overview of several depression measures, some measures were chosen based on their widespread usage listed in alphabetical order and divided into two categories—clinician ratings and self-report inventories [12, 13]. However, purely relying on self-report questionnaires also limited the availability and effectiveness of today’s mental health service.

With current technological developing, it can provide many methods for continuous monitoring the individuals of the psycho emotional status [14]. Automatic depression recognition researches, as part of inchoate assessment or relapse prevention programs, aims to provide reliable indices of stress-related risk [15, 16]. An additional as natural, easily observed body activity, human action has been found to reflect patients’ mental status, including the state of depressive disorders [17, 18]. For depression evaluation, head movement analysis has been extensively used or body expressions, gestures and head movements could be as significant as the typical symptoms of depression. Depressive state was reflected in low energy, slow movement and expanded limbs and torso [19, 20]. Normally human activity like walking, researches keep a attention on arm swing and vertical head movements reducing, reduced walking speed, abnormal hand movements and head position in walking comparing to neutral, larger lateral swaying movements of the upper body and a more slumped posture and depressed patients showed larger reaction time variability [21, 22].

Existing methods can be classified as relying on either upper torso or relative limbs part movements [23, 24]. Relative body part skeletal movements represent orientation and displacement can be captured and extracted via Kinect [25, 26] from the sensor’s origin expressed in space coordinates. With the advantage of high performance and cost portability and low cost, Kinect may be a practical option to conveniently record body gestures in a variety of disease detection studies [27, 28]. High qualified and efficient computational models would be built which could recognize depression based on kinect-recorded skeleton data, rather than only find some motion gesture features relevant to depression [29]. Using Kinect for gait analysis can provide a contactless and low-cost method for depression recognition [30, 31]. Using machine learning methods to automatically recognize the un-depression and depression, these original data driven features could not provide a high-level description of the gesture pattern of depression, such as turning, arm swing, etc., but may involve more potential information which would be calculated for recognition [32]. In general, because shaking or fidgeting behavior, psychomotor agitation or retardation, and diminished ability to concentrate have been considered as signs of depression, the whole body, the upper body, or separate partial body [33] involved in body gestures can contribute to the depression assessment.

**Fig. 1:**
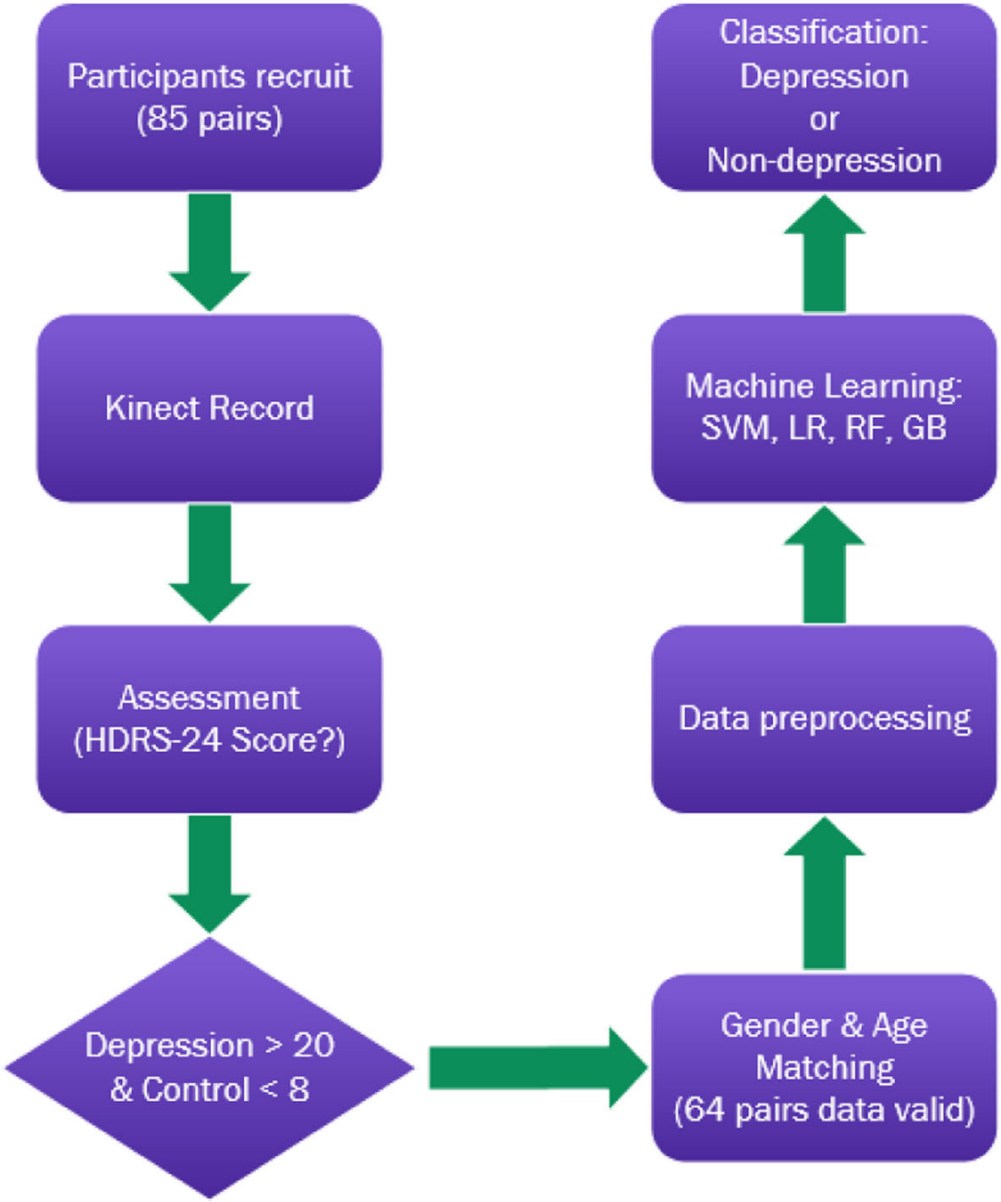
Skeleton data based depression recognition

Discussed above topics of the present research, but few approaches have exploited their applications, which could be an objective, easily accessible data source, stimulation tasks standardization of depressive state detection method hasn’t been fully built up yet. In this paper, the procedure shown in Fig. 1, this proposed experiment focus on body language cues generated by human smiple action, and briefly reviewed the excellent relevant methods in the kinect body capturing channel according to the feature extraction and preprocessing, stimulus tasks design, handcrafted dataset based and using machine learning methods for depression detection.

## Methods

### Participants

The collection job was completed in the special lab of Shandong Mental Health center. In this study, the dataset contains 85 depression patients from the Shandong Mental Health center and 85 non-depression person as control group recruited from society on aged 18 to 65. There were 170 participants (85 pairs) in this experiment, which everyone would be accessed by psychiatrists referred to the HAM-D assessment standard [34]. Although there was subjective judgment of psychiatrists in the scoring standard, it must be guaranteed that all the subjects’ scale scoring process is completed by the same psychiatrist with rich experience, so as to eliminate the influence of these subjective factors as much as possible. Participants would be led into a room that is setup to keep interaction with researchers to perform the stimulus task and allowed them to feel comfortable in the experimental surroundings. Experiments protocol has obtained permission from the Shandong Mental Health Center and participants.

Kinect V2 device takes advantage in its low price and depth sensing with strong and efficient computational model capability, which is used to capture the participant’s kinematic skeletal features, and it is easy to extract original sequential data. 25 human-skeleton joints coordinate stream would be recorded, which were triggered by body joints movement and involuntary swing, so sequential skeletal data generally followed the body event-indexes. According to the Kinect basic parameters, in order to capture the whole-body movement, participants were standing 3 meters in front of Kinect to complete procedure following the task direction audio covered by researchers. To improve the recognition rate of equipment and avoid the influence of illumination or disturbed information, there is a green curtain was placed behind the subjects as detection background.

### Stimulus task

Considering some patients’ conditions and current status, psychiatrists explained the procedure to the participants before starting. There are many articles about human body emotion expression [35–37], but few talked about the design standard of stimulus task for depression detection, so this series of simple action was designed based on the advice of psychiatrists and considering the physical condition of some depression patients. According to the psychiatrist’s description, even if the age standard for recruiting subjects were set to be 18 to 65 years old, because some patients may be under the long-term influence of depression, some patients’ motor function has also been greatly affected, so it is not easy to make more complex movements. To eliminate irrelevant influence like education, age, profession, gender et.al, the stimulus task in this experiment should also make the limb move much larger, so as to facilitate Kinect better recognition. All of the participants would follow the action direction, standing on the specified location. Stimulus task was separated into five part, which all the participants were asked to lift two-hands, lift left hand, lift right hand, turn right, turn left and reset without intentional previous training, lasting 60 s by Kinect continuously recording in order to acquire adequate high-quality body kinematic data.

### Inclusion and exclusion

Depression patients’ recruitment was perhaps the most challenging job of this research, after evaluating by the professional psychiatrists, which they would be pre-screened according to their treatment condition. Hamilton Depression Rating Scale (HDRS) [38], which is used the version contained 24 items (HDRS-24) in this experiment, provide an abbreviated indication of depression and a guideline for recovery assessment, which includes a multiple item questionnaire. It has been criticized for clinical practice using as it places more emphasis on insomnia than on feelings of hopelessness, self-destructive thoughts, suicidal cognition and actions, and the total score is compared to the corresponding descriptor.

Through mood, suicide ideation, insomnia, anxiety, agitation or retardation, feelings of guilt, weight loss, and somatic symptoms et.al judging, the questionnaire is used to evaluate the severity of their depression, which is designed for adults’ assessment. Some of the patients was screened who got score <20, as psychiatrists analysed that they recovered by hospitalization in the ward, while they had been treated for more than two weeks in particular. Dataset inclusion criteria was set as depression >20 and control <8, then 64 pairs of data were selected from 85 pairs of total recruited samples following age and gender absolutely matching principle. There were 3 handcrafted datasets in this experiment, which were sorted dataset (64-pairs), gender based dataset (29male and 35female), age based dataset (32 pairs of age >= 40 and 32 pairs of age <40). Aging seems to be a critical reason of human capacity for human action ability limitation, according to the principle of gender and age totally matching, a total of 64 pairs (128 samples) of valid data were screened. The baseline age of participants was set form 18 to 65, and average age is 37.61 (std=14.71). The depression group average HDRS-24 score is 29.70 (std=0.84), where control average score is 0.66 (std=1.24). Statistical details shown in Table 1.

**Table 1:** HDRS-24 score statistics of participants

Age	Average age	Count(pairs)	Proportion/total	% Female	Score(Depression)	Score(Control)
18–19	18.67	6	9.38%	50.00%	32.67	0.00
20–29	24.25	12	18.75%	66.67%	30.83	0.33
30–39	34.44	13	20.31%	38.46%	34.23	1.00
40–49	44.64	14	21.88%	71.43%	25.38	1.50
50–59	54.21	15	23.44%	53.33%	28.80	0.87
60–65	62.75	4	6.25%	25.00%	30.50	1.75
<=40	27.50	32	50.00%	50.00%	29.69	0.59
>40	51.50	32	50.00%	59.38%	27.78	1.22
male	40.62	29	45.31%	-	28.83	0.90
female	38.80	35	54.69%	-	28.92	0.91
Total(18–65)	39.63	64	-	54.69%	28.88	0.91

### Data extraction

Using the Kinect-default 3D coordinates with the sensor position as the initialization may cause non-negligible deviation in the stimulating progress, due to the different positions relative to Kinect camera of different participants during recording participants response. Although the recorded Kinect file contained much more information, the solely Kinect-skeletal modality was used in this research work, as this may be all that was available for depression detection. The skeleton data recordings of participants activity from Kinect was the 3-dimentinal accelerations of the 25 body key joints. Human torso is the most reliably detected area, even under heavy occlusions, as it can be accurately estimated based on other features’ 3D positions. Using the rigid transformation obtained from the calibration, the skeleton sequence of limb movement is mapped to Kinect original coordinate space. Kinect will capture human skeleton joints coordinate space as sequential data, so the original data can be extracted from the recorded file. The extracted data is the spatial position (X, Y and Z axis) of each joint generated by all frames of Kinect during the stimulation task. In order to facilitate the data extraction work, we developed a Kinect-record file extracted tool based on.net core 3.0 platform.

### Data preprocessing

Before feature mapping, we noticed that the skeletal data are flexible and variant in the sequence, which causes great difficulties in joints relationship and decisive kinematic information analysis. In more complicated cases, normalization referred to more sophisticated adjustments where the intention is to bring the entire probability distributions of adjusted values into alignment. In the case of normalization of scores in depression assessment, there may be an intention to align distributions to normal distribution. Different approaches to normalization of probability distributions is quantile normalization, where the quantiles of the different measures were brought into data standardize. Data normalization method was used for data preprocessing as the below equation:

1 $$ \mathrm{x}_{\mathrm{i}}^{*}=\frac{x_{i}-x_{\min }}{x_{\max }-x_{\min }}  $$

Feature scaling is used to bring all values into the range [0,1]. The stimulus task duration is about 60 seconds. Because of the Kinect human skeleton recognition mechanism and participant’s performance, each participant’s skeletal data length is different even in the same task. Using python numpy padding ‘0’ method for data length matching, the further processed data could be fed into the machine learning model directly.

### Classifiers

Four state-of-the-art machine classifiers [39–41] are used for depression classification: Support Vector Machines (SVM), RandomForests and Gradient boosting. these ML methods were applied in the kinematic skeletal based depression classification. The classification models separate subjects into two groups: depression and non-depression. Experiments were conducted on the handcrafted skeleton dataset, which were separated 80% for training and 20% for testing. The precision, roc-curve, recall, sensitivity, specificity, confusion matrix were calculated as the measurement of methods, which were commonly used to evaluate classification methodologies.

#### Support vector machine

Support vector machine is a kind of supervised learning model with associated learning algorithms that performs pattern classification by finding a decision processor that enables classification. Given the set of training examples, each marked as belonging to two categories (depression and non-depression), trained SVM algorithm builds a model that assigns new feed sample to predicted category. SVM can efficiently perform a non-linear classification using what is called the kernel trick, implicitly mapping their inputs into high-dimensional feature spaces. SVM has been integrated in sklearn, one strong machine learning python library, and the decisive parameters like, C: the penalty coefficient, kernel: the kernel type, gamma: kernel coefficient are set as C: 9, kernel: radial basis function (rbf), gamma: 0.69. And the radial basis function is defined as:

2 $$ K\left(\mathbf{x}, \mathbf{x}^{\prime}\right)=\exp \left(-\frac{\left\|\mathbf{x}-\mathbf{x}^{\prime}\right\|^{2}}{2 \sigma^{2}}\right)  $$

where ∥**x**−**x**^′^∥^2^ is like the squared euclidean distance between the two feature vectors and *σ* is a free parameter.

#### Logistic regression

Logistic regression is a statistical classification model uses a logistic function to model binary dependent variable. Mathematically, the binary logistic model has a dependent variable with two possible values, such as depression/non-depression which is represented by an indicator variable, where the two values are labeled "0" and "1". The important parameters of logistic regression like C: inverse of regularization strength, penalty: specify the norm used in the penalization, tol: tolerance for stopping criteria, are set as C: 9, penalty: l2 regularization formulation, tol: 0.001.

#### Random forest

Random forests or random decision forests are an ensemble learning method for classification task that operate by constructing a multitude of decision trees at training time and outputting the class that is the mode of the classes mean/average prediction of the individual trees. Random forest classifier is a meta estimator in this research that fits with n_estimators: 300 of decision tree and criterion: function to measure the quality of a split with gini impurity.

#### Gradient boosting

Gradient boosting (GB) is a machine learning method for classification problems, which produces a prediction model in the form of an ensemble of prediction models. It builds the model in a stage-wise fashion, and it generalizes them by allowing optimization of an arbitrary differentiable loss function. GB method actually adopts the addition model and the forward distribution algorithm following the equation:

3 $$ \hat{f}(x)=f_{M}(x)=\sum_{m=1}^{M} \sum_{j=1}^{J} c_{m j} I\left(x \in R_{m j}\right)  $$

where *M* is the maximum number of iterations and *j* is the leaf node region of the *m*_*th*_ tree. GB performs binary classification with special case where only a single regression tree is induced, and the crucial parameters like n_estimators: the number of boosting stages to perform, learning rate (lr):shrinks the contribution of each tree, loss: loss function to be optimized are defined as n_estimators: 300, lr: 0.03, loss: deviance.

## Results

### Performance of predictive classification models

**Fig. 2:**
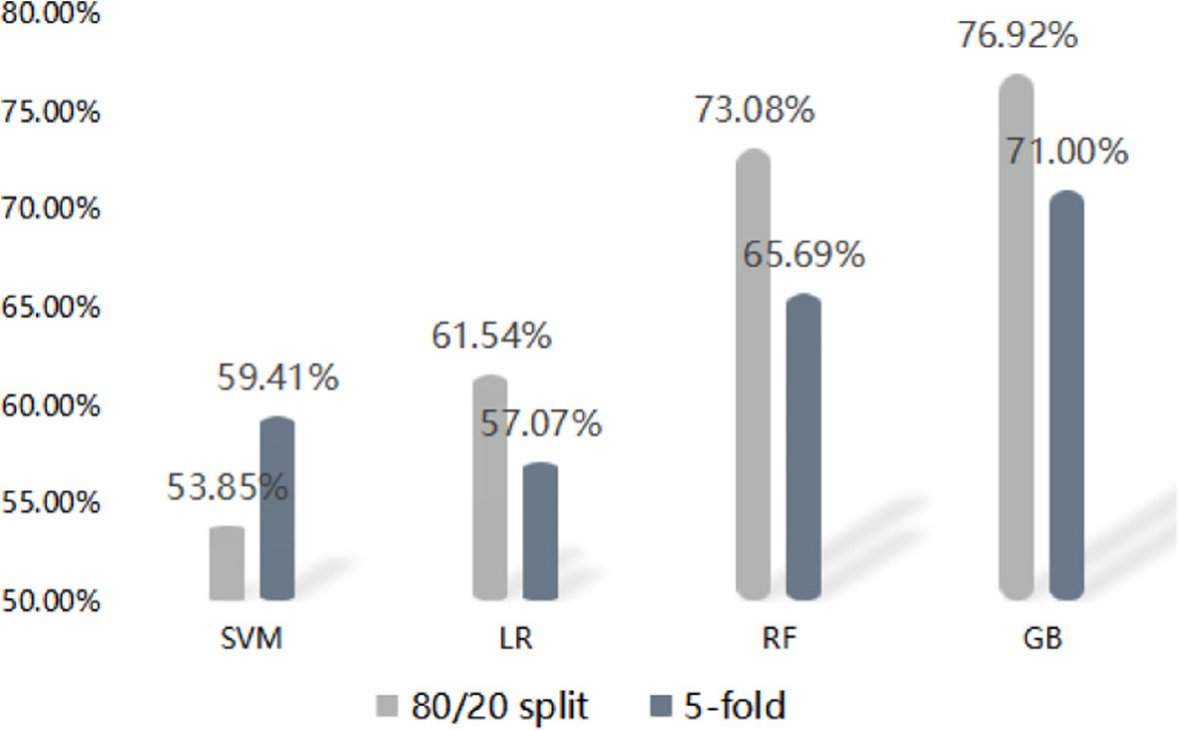
The presision of 80/20 cross-validation and 5-fold

Table 2 shows the results of the classifiers classification of patients and healthy controls based on the sequential skeletal data. As can seen, the Gradient Boosting get accuracy of 76.92%, compared to other three machine learning methods (SVM, LR, RF). Using each of the four machine learning classifiers, the best model (GB) obtains with considerable predictive signal AUC is 0.90. In general module evaluation, sensitivity and specificity are very important statistical measures of the performance in binary classification task. Specificity describes the proportion of true positives (Depression group) that are actually identified, and sensitivity expresses the proportion of true positives (Control group) that are correctly identified. GB reaches the best performance in methods with specificity of 78.57% and sensitivity of 75.00%. Classifiers have performed experiments on 5-fold method to segment training and test data trained 30 epochs on four Machine Learning method this manuscript mentioned, and Gradient Boosting (GB) got the best performance 71.00% accuracy. Except SVM method got higher accuracy on 5-fold cross-validation, other three methods performed worse, shown in the Fig. 2. Although the best performance GB only get lower accuracy than on 80/20 cross validation, it was still in a reasonable range.

**Table 2:** Results of classifier

Classifier	Accuracy	Precision	Sensitivity/Recall	Specificity	AUC
SVM	53.85%	60.00%	66.67%	50.00%	0.54
Logistic Regression	61.54%	66.67%	66.67%	57.14%	0.54
Random Forest	73.08%	73.33%	76.92%	69.23%	0.82
Gradient Boosting	76.92%	78.57%	78.57%	75.00%	0.90

**Fig. 3:**
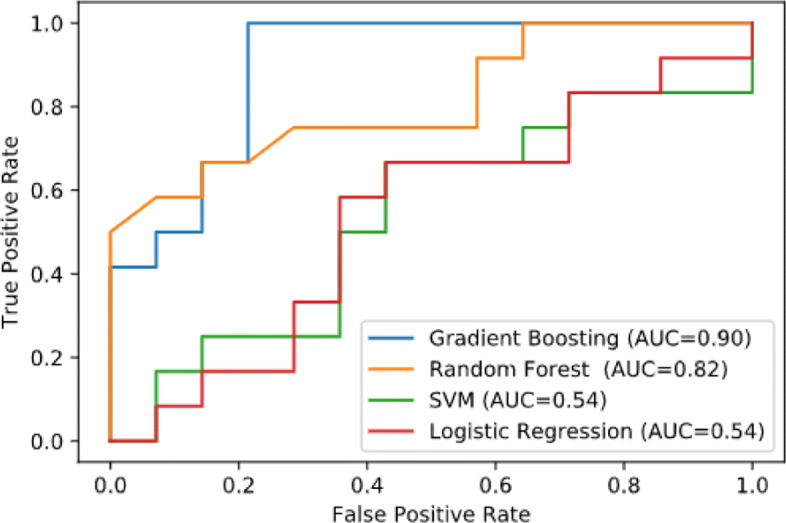
ROC curve of Classifiers

Plots of the four methods’ result above in the ROC space are given in the Fig. 3. The result of method GB clearly shows the best predictive power among RF, LR, and SVM. The closer a roc curve from a contingency table is to the upper left corner, the better it performs, but the distance from the random guess line in either direction is the best indicator of how much predictive power a method has. If the curve is below the line and is closer to the diagonal, all of the method’s predictions must be reversed in order to utilize its power, thereby moving the result above the random guess line. The result of SVM lies a little better on the random guess line, and it can be seen in the table that the accuracy of SVM is 53.85%. In these methods, the GB performs the best prediction capacity, and it is also reflected in its recognition accuracy, which reaches 76.92%.

### Gender-based classification

**Fig. 4:**
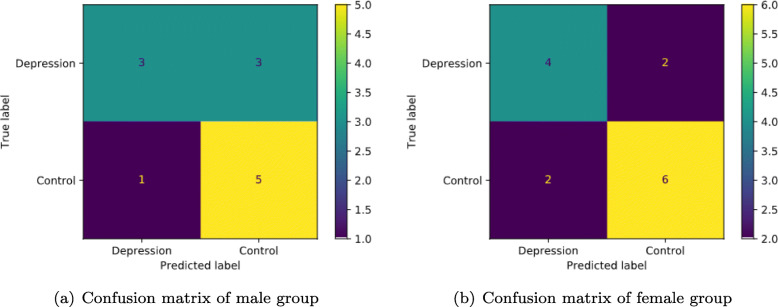
Confusion matrix of gender-based

Confusion matrix is used to specific table layout that allows visualization predicted result of the best classifier Gradient Boosting. As is shown in Fig. 4, row of the matrix represents the instances in a predicted class while each column represents the instances in an actual class, so other measures like sensitivity et.al is very easy to be calculated. In order to indirectly compare the ability of the best performance method GB to make prediction on gender based classification, this research handcrafted two datasets by gender, which included 29 pairs samples in male subset and 35 pairs samples in female subset. The best result of gender based depression classification is as shown in the confusion figure. In the male group, some key evaluation indexes are listed, e.g. depression prediction accuracy 66.67%, sensitivity 75.00%, specificity 62.50%, recall 75.00%. There are 12 testing group samples in the male group, the model judged that 3 were depression items, and of the 6 total items depression group, it predicted that 3 were non-depression (Control). In the female group, the depression prediction accuracy is 71.73%, sensitivity 70.83%, specificity 71.43%, recall 70.83%. There are 14 in the female testing group, the model judged that 4 were depression items, and of the 6 total items depression group, it predicted that 2 were non-depression (Control). Excluding the factor of balanced sample distribution, the average accuracy of male group is still higher than that of female group.

### Age-based classification

**Fig. 5:**
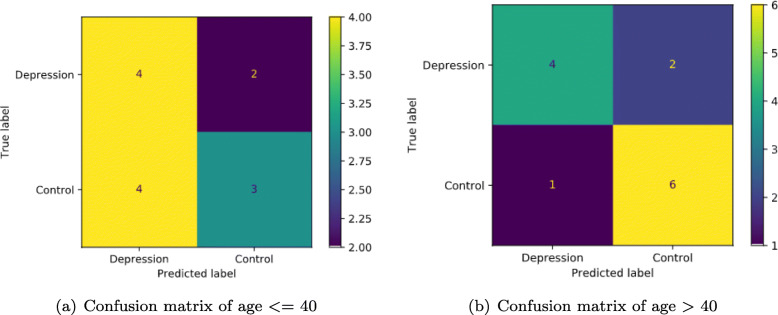
Confusion matrix of age-based

In this classification task, the best performance method GB was still applied to make prediction on age based classification, and two datasets were separated by age (age >40 and <= 40), coincidentally, both groups included 32 pairs of samples. The best result of age based depression classification is as shown in the confusion Fig. 5. From the HDRS-24 score statistical Table 1, depression class score of age <= 40 group was 29.69 less than the group of age >40. But the results are in a huge gap, the second group of age >40 result is obvious better than the first group. Though the number of samples of the two groups is the same, the recognition accuracy of age <= 40 group is higher than that of age >40 group. In the test set, the two groups both contained 13 samples. In the age <= 40 group, some key evaluation indexes are listed, e.g. depression prediction accuracy 53.85%, sensitivity 50.00%, specificity 60.00%, recall 50.00%. In the age >40 group, the depression prediction accuracy is 76.92%, sensitivity 80.00%, specificity 75.00%, recall 80.00%. The precision of the elderly group is significantly higher than that of the young group.

## Discussion

**Fig. 6:**
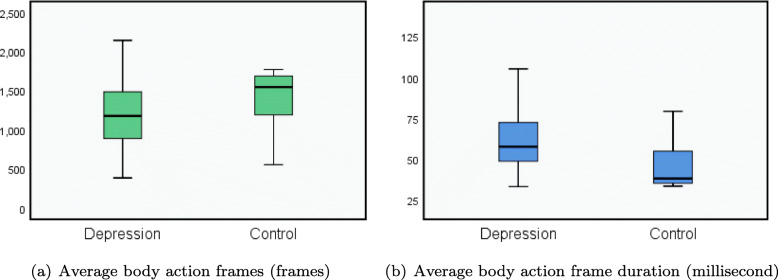
Body action frames and duration in two groups

Kinect V2 device is very sensitive to human body activity. That’s mean more frequent and continual body joints movement and involuntary swing will invoke more sequential skeletal data generated. As is shown in the Fig. 6, the descriptive statistics: means, standard deviations.et.al scores and evidence of consistencies for each pair of sample. Statistical analysis of the results showed that it has a significant deference (p =0.005 <0.05) of captured body action frames between depression group and control group during the completion of the stimulation task. The average action frames of depression group is 1222.81 (std=413.1), and control group is 1424.31 (std=307.22). Depression group and control group action frames were recorded by same Kinect device during the same stimulus task, but the control group average captured action frames were obviously greater than depression group, and the standard deviation of control group was smaller. Particularly, we also calculated the difference of the duration of the action frame caused by the different number of action frames triggered by the two groups in the same action task time. The average action frame duration of depression group is 54.60ms, and control group is 44.11ms (p =0.009 <0.05). Because of the recognition principle of Kinect, even in the same stimulus task, the time difference between two motion frames captured by Kinect is not a constant, which means that the frame rate is not constant. From the frame duration, the average frame rate can be calculated that depression group is 18 and control group is 23.

In addition, two indexes calculated by original data-driven measurements, the frame-duration and total frames both have significant deference, which are independent from preconceived assumptions and could make the findings more objective. In this present study, all four machine learning methods mentioned achieve the above guess prediction rate, and GB method gets the best detection accuracy. The preprocessed skeleton features are directly input into the effective predictive machine learning model, which extracted from Kinect captured participant’s body joints 3D-spatial coordinates. Considering that some extracted features may be redundant or uninformative for final prediction, strong descriptors takes advantage in depression evaluation for participant’s body movement kinematic features during the stimulus task, Limited by the integrating different dimensional features into predictive model, classifiers may not be good at understanding the differences between individual samples intuitively, such as participants’ body shape, height, however, which can cover object’s depressed status detection based on the body manifestations comprehensively reflected in stimulus task.

Result shows that ML models have achieved good performance in gender related depression recognition. The difference between male and female in the validation set is mainly due to the inconsistent data distribution. There are 29 pairs male (45.31% proportion) and 35 pairs female (54.69% proportion) in the whole dataset, and the gap is 9.38%. The precision of male group is 66.67%, and that of female group is 71.73%, and the gap is 5.06. Even considering the quantity of dataset between male and female groups, the recognition rate of male group is higher than that of female group. but the precision gap between male and female may be caused by unequal distribution of sample data. In general, the proportion of depression in genders is the difference observed, but male achieve a higher recognition rate of depression based on human posture detection [42], and our experimental results are consistent with this point. Of course, we can not deny that the accuracy rate may be affected by diverse factors, like athletic ability, the body shape difference between male/female, and the existence of special individual participants. As Dael [43] reported that head pose and movement classification results got higher accuracy on male group, which mentioned that a physical abnormality rather than a behavioural one in head movement. Men might amplify their reflecting of body movement-based stimulus task, so male are more likely to be detected than female.

In the age-based depression classification experiment, even if the number of samples in the depression group and the control group is exactly the same, the recognition accuracy shows great difference, but the average HDRS-24 score of the two groups is very similar. The recognition accuracy of the elderly group is significantly higher than that of the young group. Result shows that the GB classifier may be more suitable for older group in our experiments.

### Limitations and strengths

There are also shortcomings in this method of depression detection that recruited the subjects knew little about their history of depression, especially for the depression group, when patients with depression receive treatment or are currently receiving treatment, it may affect the recognition result of the mode. We must also note that the clinical diagnosis of depression is a very rigorous and complex process, and the current depression of a patient may not be fully reflected in the HDRS-24 scale. Therefore, through the screened sample, the individuals who were evaluated as depression, it can’t be considered as a "real" depressive patient in fact. Beside, the used classifiers still have lots of disadvantages, e.g. SVM is that data preprocessing and parameter adjustment need to be very careful, and LR is easy to under fit amd it can’t deal with many kinds of features or variables well. RF and GB like a black box, which are hard to control the internal operation of the model, can only try between different parameters and random seeds, even GB training takes longer. Table 3 listed several depression detection methods based on body posture, which achieved good detection results. Different from other methods, we use stimulus task directly like gait or head pose, which is more reflected in the fact that the subjects are always in a state of active feedback according to the task rather than passive observation. The calculation results based on the skeleton motion data show that the method is effective when confirming the severity of anxiety and depression measured by the questionnaire, The potential of this data-driven approach in depression detection is demonstrated. Based on original Kinect-3d coordinates sequential features of the participants’ 25 body joints, an effective accuracy model is established by machine learning method, especially the GB classifier, which also achieves good results in gender-based and age-based classification.

**Table 3:** Methods for depression detection

Classifier	Accuracy	Dataset description	Samples	Assessment
Alghowinem [43]	71.20%	Head pose and movement	80	Question
Zhao Nan [44]	64.00%	Gait data	179	PHQ-9
Wang Tao [30]	93.75%	Gait data	95	PHQ-9
Chunke Jing [45]	73.00%	Gait data	88	PHQ-9
Ours	76.92%	Body simple action	128	HDRS-24

In this study, the proposed a skeleton feature descriptor based on a specific direction of movement, a slight subjective evaluation (HAM-D), which limits the study integrate different forecasting capabilities. However, the kinematic features in our study do not provide sufficient evidence for participants’ reflection, which can more comprehensively cover the psychological state information reflected by the individual in the stimulation task. The experimental results show the effectiveness of the model cognitive characteristics questionnaire measures the severity of depression, and it shows the potential of this data-driven method in the field of psychometrics.

## Conclusion

In this paper, using Kinect V2 device to record participants kinematic skeleton data of the participant’s 25 body joints, the presented spatial features and low-level features is directly extracted from the record original Kinect-3D coordinates. The scored-depressed and non-depressed individuals can be well classified by computational models which were import processed data directly. Meanwhile, in the gender based depression classification also get reasonable accuracy. In particular, the recognition results of the old group are significantly better than that of the young group. All these findings suggest that kinematic skeletal data based depression recognition can be applied as an effective tool for assisting in depression analysis. In future work, we will extend research to depression severity detection for the further improvement of the overall performance.

## Data Availability

The datasets used and/or analysed during the current study are available from the corresponding author on reasonable request. Declarations
